# Multi-Targeted TKIs in Patients with Advanced Ewing Sarcoma: A Systematic Review and Single-Arm Meta-Analysis

**DOI:** 10.3390/cancers18030465

**Published:** 2026-01-30

**Authors:** Isabella Michelon, Caio Ernesto do Rêgo Castro, Ana Paula Querino Belluco, Maria Inez Dacoregio, Jonathan Priantti, Russell Gardner Witt, Steven Attia, Maysa Vilbert, Ludimila Cavalcante

**Affiliations:** 1 Department of Hematology/Oncology, University of Virginia, Charlottesville, VA 22903, USA; 2 Faculty of Medicine, University of São Paulo, São Paulo 05508-220, SP, Brazil; caioernestorc@gmail.com (C.E.d.R.C.); m.dacoregio2707@gmail.com (M.I.D.); 3 State University of Campinas, Campinas 13083-887, SP, Brazil; anapaula.belluco@gmail.com; 4 Department of Internal Medicine, Federal University of Amazonas, Manaus 69067-005, AM, Brazil; jonathanpriantti@gmail.com; 5 Department of Surgical Oncology, University of Virginia, Charlottesville, VA 22903, USA; syd3xz@uvahealth.org; 6 Hematology and Oncology Department, Mayo Clinic, Jacksonville, FL 32224, USA; attia.steven@mayo.edu; 7 Department of Medicine, MetroWest Medical Center, Framingham, MA 01702, USA; maysavilbert@gmail.com

**Keywords:** tyrosine kinase inhibitor, Ewing sarcoma, multiply refractory disease, TKI

## Abstract

**Background/Objectives**: Standard treatment of multiply relapsed Ewing sarcoma remains to be established. Recent studies evaluating tyrosine kinase inhibitors (TKIs) with anti-angiogenic properties have shown encouraging results. Therefore, we conducted a systematic review and meta-analysis to explore the efficacy and safety of TKIs in patients with Ewing sarcoma. **Methods**: We comprehensively searched PubMed, Embase, and Cochrane databases for clinical trials (CTs) and cohort studies assessing TKIs in the treatment of advanced Ewing sarcoma patients who received at least one prior line of therapy. The main outcome was objective response rate (ORR). All analyses were conducted using R software (v.4.2.2), employing random effects models with 95% confidence intervals (CIs). **Results**: We included 14 studies (seven phase II CT and seven retrospective cohorts), comprising 257 patients. The following TKIs were evaluated: cabozantinib, regorafenib, apatinib, anlotinib, sorafenib, lenvatinib, sunitinib, fruquintinib, and imatinib. In a pooled analysis of all Ewing sarcoma patients treated with TKIs, the ORR was 23% (95% CI, 11.2–37.1%) and the DCR was 61.1% (95% CI, 47.3–74.2%). Responses were numerically higher but statistically nonsignificant between clinical trials and real-world studies. The analysis including only single-agent TKIs showed better responses for anlotinib and apatinib, yet these drugs are not available in Western countries. Among the FDA-approved TKIs, superior outcomes were noted with single-agent cabozantinib. (ORR: 21.6%) and regorafenib (ORR: 11.3%). Several studies did not report toxicity data exclusively for Ewing sarcoma patients; thus, conclusions about toxicity are mostly based on the general population of studies and may not be fully representative of Ewing sarcoma patients. **Conclusions**: Anti-angiogenic TKIs have shown important anti-tumoral activity in patients with Ewing sarcoma. Efficacy was consistently seen in both clinical trials and real-world studies. Nonetheless, there are important differences in study design and population that may limit our interpretation of efficacy and toxicity findings.

## Simple Summary

Ewing sarcoma is a rare and aggressive cancer that often relapses after treatment. There is no clear standard therapy for patients whose disease progresses. Tyrosine kinase inhibitors have recently shown promising results. We reviewed and pooled data from published clinical trials and real-world studies to better evaluate the efficacy and safety of tyrosine kinase inhibitors in relapsed Ewing sarcoma patients. In our pooled analyses of 14 studies, we found an objective response rate of 23% and a disease control rate of 61.1%. Cabozantinib and regorafenib showed the most consistent benefits among drugs available in Western countries. These findings suggest the potential of tyrosine kinase inhibitors in the treatment of such a challenging population.

## 1. Introduction

Ewing sarcoma is a rare, aggressive primary bone malignancy frequently affecting individuals in their second decade of life [1]. It often affects lower extremity long bones and flat bones of the pelvis, and a smaller number arise in soft tissues (extraosseous). Micrometastases are found in most cases at diagnosis, and distant metastasis in up to 25% of patients, predominantly in the lung, bone, liver, and brain [2,3,4].

The standard treatment approach for patients with localized Ewing sarcoma is multi-modality therapy, consisting of chemotherapy followed by either radiation or surgery and adjuvant chemotherapy. In this setting, cure rates are higher than 70%. Less favorable rates are seen for metastatic disease. For this subset of patients, aggressive multimodality therapy with local treatment is an often-used strategy in the first-line setting, but recurrent disease is observed in up to 80% of patients with metastatic Ewing sarcoma at diagnosis [5,6]. A retrospective study with data from the National Cancer Institute found that recurrence after two years from diagnosis was the only predictor of improved survival for patients with relapsed/progressive Ewing sarcoma [7]. The estimated median overall survival was approximately 7 months in this study. Thus, patients with relapsed or recurrent disease have a particularly poor prognosis, and there is an urgent need for better strategies to address this population [8,9].

In recurrent or primary refractory disease, the choice of therapy relies on several different factors, including prior treatment, relapse-free interval, and site of recurrence. Topoisomerase I inhibitor-containing regimens are often used for initial recurrences [10,11,12]. However, survival is still limited and the optimal strategy is yet to be established, especially in the multiply relapsed setting. Thus, targeted therapies, including tyrosine kinase inhibitors (TKIs) and other investigational agents, have increasingly been evaluated.

Aberrant angiogenesis and other abnormal signaling pathways have been shown to play a key role in preclinical models of Ewing sarcoma [13,14]. Activation of the endothelial growth factor receptor (VEGF/VEGFR) axis and downstream cascade is associated with tumor proliferation and invasion, and metastatic spread properties, which, combined, result in an immunosuppressive tumor microenvironment and aggressive behavior. This provides the rationale for targeting angiogenesis pathways in Ewing sarcoma [8]. Early phase studies of multiple small-molecule TKIs that target VEGFR and other kinases demonstrated meaningful activity and acceptable safety in bone tumors, including Ewing sarcoma, and have been evaluated in multiple later-phase clinical trials and retrospective studies in the refractory setting [15,16].

Nevertheless, there is currently no standardized therapy for the multiply recurrent setting and a lack of systematic evaluation of the activity of these drugs. We conducted a comprehensive systematic review and single-arm meta-analysis to compile available evidence of different TKI agents (as single-agent or combined regimens) in the treatment of patients with refractory Ewing sarcoma.

## 2. Materials and Methods

### 2.1. Registration

This systematic review was performed in accordance with Cochrane recommendations and the Preferred Reporting Items for Systematic Reviews and Meta-Analysis (PRISMA) guidelines [17]. The PRISMA checklist for the paper and abstract is available in Table S1. The study was prospectively registered in the International Prospective Register of Systematic Reviews (PROSPERO) under the protocol number CRD42024512988.

### 2.2. Eligibility Criteria

Inclusion in this meta-analysis was restricted to studies that met all the following eligibility criteria: (1) randomized or non-randomized clinical trials, and retrospective cohort studies; (2) participants included patients with advanced, recurrent, or metastatic Ewing sarcoma; (3) treated with TKIs; (4) who had received at least one prior line of therapy; (5) and reporting any of the outcomes of interest. Studies that comprised patients with extraskeletal Ewing sarcoma were considered for inclusion. No restrictions were applied to study location and ethnicity. Only English-written reports were assessed.

We excluded studies that (1) lacked data specifically for the Ewing sarcoma subgroup; (2) lacked the population or outcomes of interest, and (3) had fewer than 5 patients.

### 2.3. Search Strategy and Data Extraction

We systematically searched PubMed, Embase, and Cochrane databases for studies that met the inclusion criteria. The search was last updated on 6 June 2025. The search used in each database is detailed in Table S2.

Two authors (CC and AB) independently screened all reports by title and abstract and selected studies for full review. The authors (IM and CC) extracted data from selected studies utilizing a standardized data extraction sheet. Disagreements were resolved by a third author (LC). Authors of the included studies were contacted in case of missing or potentially inconsistent information.

### 2.4. Endpoints and Subgroup Analysis

The efficacy endpoints of interest were (1) objective response rate (ORR); (2) disease control rate (DCR); and (3) median progression-free survival (mPFS). Overall survival was unavailable in most studies and thus was not analyzed. Safety outcomes of interest included grade ≥ 3 adverse events (AEs). We performed prespecified subgroup analyses of study design and different TKIs used as single-agent and single-agent vs. combined therapy. We also performed a meta-regression according to the median number of prior lines of therapy. The definitions of each endpoint and the assessment criteria used by each study are described in Table S3.

### 2.5. Quality Assessment and Sensitivity Analyses

We used the Methodological Index for Non-Randomized Studies (MINORS) for quality assessment of individual non-randomized single-arm clinical trials [18]. Retrospective studies were assessed through the adjusted Joanna Briggs Institute Critical Appraisal Checklist for Cohort Studies [19]. Three authors (IM, CC, and MD) independently performed the bias assessment of included studies. To assess the influence of each study on the overall effect and heterogeneity, we ran sensitivity leave-one-out analyses.

### 2.6. Statistical Analysis

Proportional meta-analyses were carried out using the number of events per total population with generic inverse variance models. We applied transformation of data as needed: logit when studies reported extreme proportions and Freeman–Tukey double-arcsine transformations when dealing with zero events or proportions of one. We reported data as percentages with 95% confidence intervals (CIs). The Cochrane Q chi-square test and I^2^ statistics were used to report heterogeneity. We pooled median PFS using standard errors (SEs) and log-transformed media PFS. The SE was obtained by converting the lower and upper CI into log scale and estimating the difference between them. We then applied the normal distribution’s scaling factor for 95% CIs. Studies were excluded from this analysis if they lacked median survival time or in which the upper or lower 95% CI was not reached. OS data was insufficient to pool for analyses. All analyses were conducted using R software (R Foundation for Statistical Computing, Vienna, Austria, v4.2.2).

## 3. Results

### 3.1. Characteristics of Included Studies

**Figure 1:**
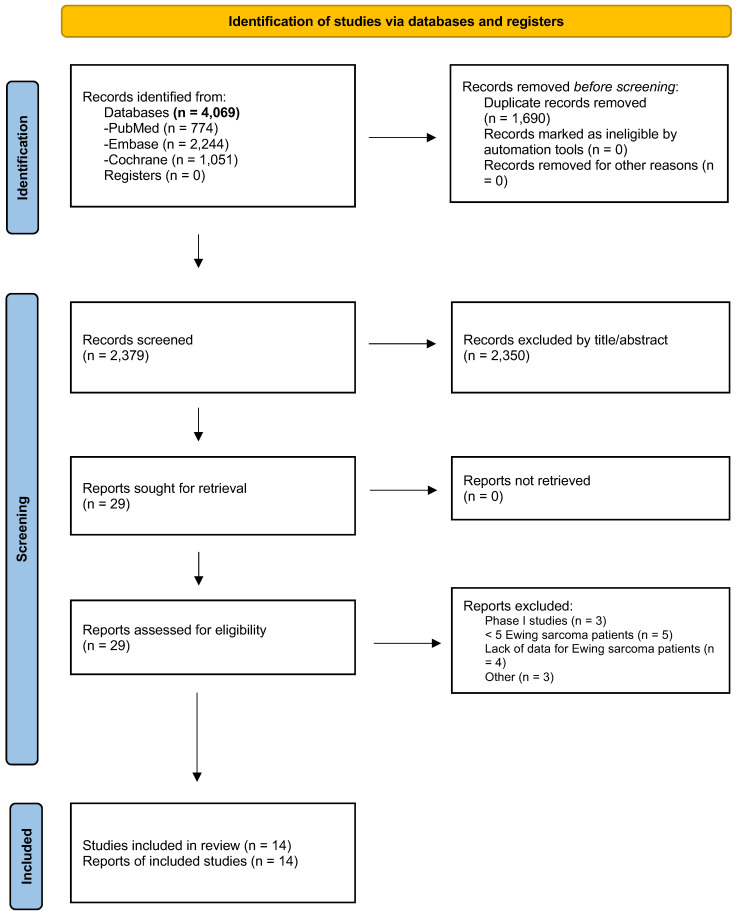
PRISMA flow diagram of study screening and selection.

The initial literature search yielded 2379 results, of which fourteen studies (seven retrospective cohorts and seven clinical trials) met our eligibility criteria (Figure 1) [15,16,20,21,22,23,24,25,26,27,28,29,30,31]. A list of studies excluded after full review is shown in Table S4.

A total of 257 Ewing sarcoma patients were included. TKIs were used as single agents in eight studies (n = 150) and combined with other treatments in six (n = 107). The TKIs used were as follows: cabozantinib (three studies, n = 66); regorafenib (two studies, n = 53); anlotinib (two studies, n = 44); apatinib (two studies, n = 21); fruquintinib (one study, n = 28); sorafenib (one study, n = 14); imatinib (one study, n = 13); lenvatinib (one study, n = 10), and sunitinib (one study, n = 8). In most studies, patients were heavily pretreated with a median number of 2 or more prior lines of therapy. Further information about each study can be found in Table 1.

### 3.2. Efficacy Outcomes

**Figure 2:**
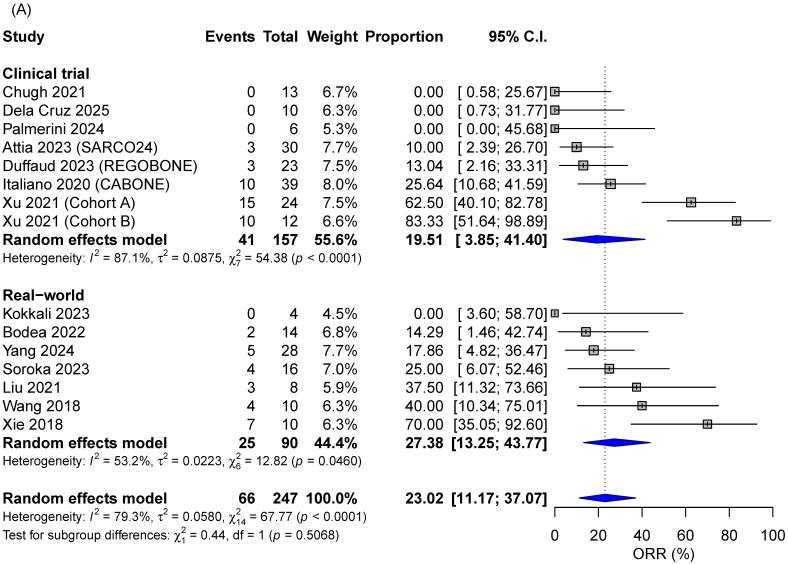

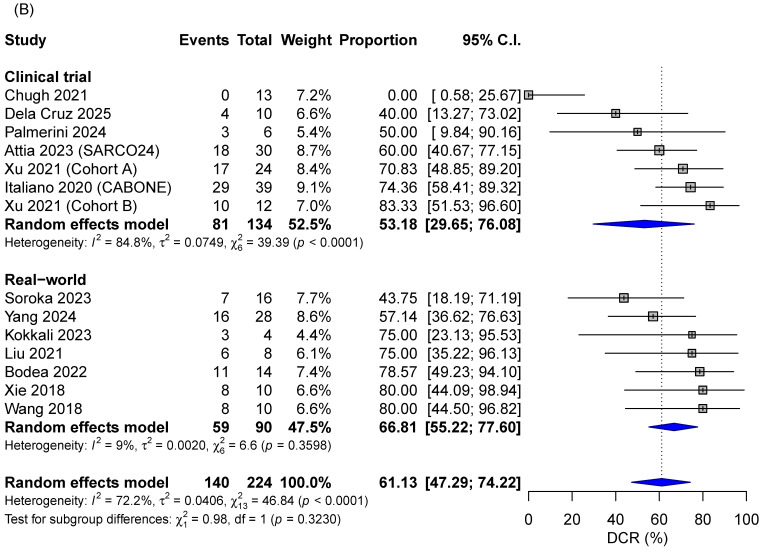
Efficacy outcomes according to study design: (**A**) ORR and (**B**) DCR. Proportions for each study are represented by a square, and the horizontal line crossing the squares indicates the 95% confidence interval. Diamonds represent the estimated overall effect of the meta-analysis based on random effect. CI: confidence intervals; DCR: disease control rate; ORR: objective response rate.

The overall ORR was 23% (95% CI 11.2–37.1) and DCR was 61.1% (95% CI 47.3–74.2), considering both single-agent and combined regimens (Figure 2). For both endpoints, no significant differences were detected considering clinical trials versus real-world studies (*p* > 0.05; Figure 2). In the subanalysis considering TKI agent used, greater responses were observed for patients receiving anlotinib or apatinib, followed by cabozantinib and regorafenib (Figure S1). Yet, patients treated with anlotinib and apatinib were less heavily pretreated, with a median number of prior therapies ranging from 1 to 1.5. On the other hand, patients on cabozantinib and regorafenib had received a median of 3 to 4 prior treatment lines.

The subanalysis considering only single-agent TKIs for ORR and DCR revealed similar findings (Figure S2). Patients on single-agent anlotinib, apatinib, cabozantinib, or regorafenib achieved a DCR superior to 55%. Responses according to drug were as follows: anlotinib (n = 8; ORR: 37.5%; DCR: 75%), cabozantinib (n = 59; ORR: 21.6%; DCR: 64.4%), apatinib (n = 10; ORR: 70%; DCR: 80%), regorafenib (n = 53, ORR: 11.3%; DCR: 60%), and imatinib (n = 13, no patients achieved ORR or DCR). The response rate was numerically higher but not significantly different for TKI-combined regimens compared to single-agent TKIs (Figure S3). Table 2 summarizes response outcomes of our meta-analysis.

Overall median PFS was 4.7 months (95% CI 3.5–6.2 months), with anlotinib showing the longest PFS of around 9 months (Figure S4). The meta-regression according to the median number of prior therapies revealed a statistically significant decrease in response rates of patients receiving TKIs as a second- or third-line treatment compared to first-line (Figure S5). Yet, this association was weak and should only be seen as exploratory. Moreover, only about 14% of the heterogeneity seen in our main ORR analysis can be related to this variable.

### 3.3. Safety Outcomes

The most common adverse events reported in each study are shown in Table S5. All studies graded AEs according to the National Cancer Institute Common Terminology Criteria for Adverse Events. In most studies, data was not specifically described for patients with Ewing sarcoma, which limits the interpretation of this data. Table S6 displays toxicity data exclusively for patients with Ewing sarcoma available across five studies. AEs varied according to the different TKI used. Regorafenib and cabozantinib appear to be often associated with electrolyte disturbances and gastrointestinal and hepatotoxicity, whereas fruquitinib was associated with hematotoxicity and kidney dysfunction. Apatinib was also associated with gastrointestinal events and skin reactions. Head-to-head comparisons of the toxicity rate among different TKIs were not possible due to the single-arm design of most of the included studies.

### 3.4. Quality Assessment and Sensitivity Analyses

Details about the tools and criteria used to analyze the quality of studies included in this meta-analysis are described in Table S7. Most prospective clinical trials were judged at moderate risk of bias for failing to meet the criteria of blinded outcomes assessment (Table S8A). Due to the nature of real-world data and unadjusted study design, retrospective studies are prone to confounding factors that could directly and indirectly mislead outcomes. Most retrospective studies included in this meta-analysis did not control for confounding factors. Therefore, they were judged at moderate risk of bias (Table S8B).

In the sensitivity analysis for ORR, a similar effect size and heterogeneity persisted when leaving each study out (Table S9). The DCR leave-one-out analysis followed a similar pattern, except for when leaving the study by Chugh out [25]. In this case, the heterogeneity drastically dropped. This could be related to several factors, such as the TKI used, study design, number of patients, and other unidentified factors.

## 4. Discussion

In this systematic review and comprehensive meta-analysis of 14 studies and 257 Ewing sarcoma patients on TKIs, the overall ORR and DCR were 23% and 61%. The subanalyses comparing clinical trials with real-world studies revealed numerically higher but statistically nonsignificant response rates for the latter. Similar responses were seen between TKI-combined regimens and monotherapy. The analysis including only studies evaluating single-agent TKIs showed better responses for anlotinib and apatinib, followed by cabozantinib. The analysis including different TKIs used revealed an overall median PFS of 4.7 months.

Currently, the standard of care for Ewing sarcoma patients with relapsed disease involves topoisomerase I inhibitor-based regimens, such as irinotecan–temozolomide (IT) and cyclophosphamide–topotecan (CT) [32]. For the former, efficacy data is primarily based on real-world results. The integrated analysis of retrospective studies by Wang et al. found an ORR of 41% and DCR of 66% for relapsed Ewing sarcoma patients on irinotecan plus temozolomide [32]. The phase III randomized rEECur trial, the largest randomized controlled trial for recurrent or refractory Ewing sarcoma to date, initially demonstrated greater efficacy for CT over IT, the latter being dropped due to lower ORR, PFS, and OS compared to CT and high-dose ifosfamide (IFOS). Ultimately, high-dose ifosfamide was proven superior to cyclophosphamide–topotecan and other tested regimens (IT, gemcitabine–docetaxel, and carboplatin–etoposide), with a median EFS of 5.7 months and median OS of 15.4 months, and was considered the preferred regimen [33]. Unfortunately, limited options are available for patients progressing or relapsing following these second-line regimens.

Several systemic therapies have been studied but most have failed to show notable activity in the multiply refractory setting. Nonetheless, TKIs have been successfully used in patients with certain sarcomas harboring tyrosine kinase receptor alterations. These receptors play a role in several essential pathways, including cell growth, angiogenesis, and survival. This provided the rationale to test these agents in Ewing sarcoma patients, with encouraging results so far. In our meta-analysis, anlotinib and apatanib elicited higher responses as monotherapies. Yet, most studies were retrospective cohorts on patients less heavily pretreated and were performed exclusively in Asia, whereas studies on cabozantinib and regorafenib were mostly clinical prospective trials. The differences in study design and populations data should be interpreted carefully. Moreover, anlotinib and apatinib are not currently available in Western countries. The FDA-approved TKIs or ones under investigation in the United States or European countries for Ewing sarcoma patients include regorafenib, cabozantinib, and imatinib. The latter targets primarily KIT and PDGFR mutations. As these are uncommon in Ewing sarcoma, this TKI has shown limited efficacy in Ewing sarcoma. In our meta-analysis, only the phase II trial by Chugh et al. evaluated this agent. No Ewing sarcoma patient achieved an ORR, and only one had a DCR.

Based on the current level of evidence, TKI-based treatment decisions are between regorafenib and cabozantinib in Western countries. Regorafenib is a multi-kinase inhibitor targeting various angiogenic, stromal, and oncogenic receptors, including VEGFR, PDGFR, FGFR, and KIT. In this meta-analysis, the two phase II clinical trials evaluating regorafenib reported a consistent ORR of around 10% for the subgroup of Ewing sarcoma patients. Median PFS ranged from 11.4 to 14.8 weeks. Cabozantinib also has a broad range of targets, but its activity is mainly attributed to blockade of the MET/VEGF axis, a pathway often implicated in sarcoma progression. Both the phase II CABONE trial and the real-world CanSaRCC study found notable responses of approximately 25% associated with this TKI in the Ewing sarcoma cohort. Median PFS was 4.4 and 3.4 months, respectively.

Regarding toxicity, both agents are associated with high rates of gastrointestinal events. Skin reactions are more commonly seen with regorafenib, whereas cabozantinib more often leads to fatigue. In the SARC024 trial, most regorafenib-related AEs were grade 1–2 hand–foot skin reactions, nausea/vomiting, and oral mucositis. Hypophosphatemia was a common high-grade event, occurring in 20% of patients. Out of 30 patients, 16 (53.3%) required dose reductions of regorafenib. In the CABONE trial, the most common low-grade cabozantinib-related AEs were fatigue, diarrhea, and mucositis. Hypophosphatemia was also the most common high-grade AE (11.1%), and roughly 20% of patients required dose reductions. In this systematic review and meta-analysis, only a few studies had data specifically for patients with Ewing sarcoma and could not be pooled for analysis due to differences in the TKI used and study design. Therefore, most available data of TKI toxicity for Ewing sarcoma is based on extrapolated data from studies including a broader population of sarcoma patients, which may not be representative of the group with Ewing sarcoma.

To date, there are no head-to-head comparisons between regorafenib and cabozantinib in terms of toxicity or efficacy. The retrospective study CanSaRCC aimed to assess the activity of regorafenib and cabozantinib in relapsed/refractory bone tumors, yet their comparative analyses were limited by a small sample size. Based on evidence currently available, cabozantinib seems to be associated with higher responses and lower rates of dose reduction, although studies on other TKIs differed in design and number of patients.

Other types of agents, such as immune checkpoint inhibitors, have shown activity in sarcoma patients [34]. The phase II SARC028 trial, for instance, evaluated pembrolizumab monotherapy in over 80 patients with advanced sarcomas. Although it has shown moderate activity in some sarcoma subtypes, patients with Ewing sarcoma failed to achieve an ORR [35]. More encouraging results were seen for the combination of sunitinib plus nivolumab in the phase I/II IMMUNOSARC trial, in which over 30% of advanced bone sarcoma patients achieved a 6-month PFS [30].

Emerging therapies and novel strategies in the treatment of Ewing sarcoma now include monoclonal antibodies and alkylating agents, both showing mixed results. A recent randomized phase III trial (AEWS1221) showed increased toxicity with no survival benefit in adding the anti-insulin-like growth factor-1 receptor ganitumab to interval-compressed chemotherapy for untreated metastatic Ewing sarcoma [36]. INBRX-109 is a DR5 agonist, which induces apoptosis in tumor cells and is being studied in Ewing sarcoma in combination with temozolomide and irinotecan in the refractory setting (NCT03715933). SARC037 is a phase I/II clinical trial evaluating trabectedin combined with low-dose irinotecan in suppressing the activity of the oncogene EWS::FLI1 in relapsed/refractory Ewing sarcoma patients (NCT04067115). Their initial findings support the activity of this regimen, with 5 out of 16 patients achieving responses and a 6-month PFS of 37.7% [37]. The study was recently completed and is pending final results.

In parallel, there is increasing interest in incorporating TKIs in earlier treatment lines of patients with sarcoma [27]. The randomized noncomparative phase II REGOBONE study evaluated regorafenib in patients with Ewing sarcoma after progression on initial chemotherapy [27]. The study has not met its primary endpoint (progression-free rate at 8 weeks); however, there was a modest benefit for the 23 patients on regorafenib compared to the 13 on placebo. At 8 weeks, 13 out of the 23 patients were progression-free in the regorafenib cohort compared to only 1 patient in the placebo group. TKIs combined with other strategies may also be an alternative in earlier lines, although overlapping toxicity may be concerning. The REGO-INTER-EWI phase Ib trial is exploring regorafenib combined with conventional chemotherapy in patients with newly diagnosed multi-metastatic Ewing sarcoma (NCT05830084). Initial findings have been recently reported, and preliminary toxicity data suggest that the combination is well tolerated [38]. Further efficacy analyses are expected.

This meta-analysis highlights the efficacy of different TKIs in relapsed/refractory Ewing sarcoma patients. However, the prognosis of this population remains poor and standard universally established regimens are still lacking. Moreover, the role of TKI-combined regimens and their influence on efficacy and toxicity is still unclear. Mechanisms leading to resistance also warrant further investigation. Advances in biomarker identification tools and molecular profiling may uncover actionable targets and guide patient selection and response to treatment.

High heterogeneity was seen in our analyses. Multiple factors may have influenced this, namely, differences in study design and populations, TKI drugs of choice, and schedule and combination regimens. We thus believe that there are some unidentified factors that could influence our analysis. Yet, Barker [39] and colleagues discuss in their guide of proportional meta-analysis that high heterogeneity is expected in the scenario of single-arm estimates. It commonly arises from differences in population characteristics and reflects the nature of single-arm analysis, rather than data inconsistency. Moreover, the authors support that current tests used to assess heterogeneity in single-arm analysis (e.g., chi-squared and I^2^ statistic) were not designed for proportional analysis. In the absence of more appropriate tests, these are still reported in single-arm forest plots; they should, however, be interpreted with caution. Nonetheless, we performed leave-one-out sensitivity and subgroup analyses aiming to explore heterogeneity sources and the impact of individual studies on it.

This study has some limitations, the first being that only few studies have evaluated TKIs in Ewing sarcoma patients. Therefore, the number of studies and patients included in this meta-analysis was limited. In many of these studies, Ewing sarcoma was a subgroup within bone sarcoma patients and data was only partially available. Due to the lack of head-to-head comparisons among different TKIs, we could only estimate the percentage of responses for each TKI and draw conclusions based on indirect comparisons. Moreover, we could not explore important factors such as age and mutational status. Finally, network meta-analysis allows for comparisons among different agents within the same class. This would be the ideal approach to explore the outcomes of TKIs in patients with Ewing sarcoma. Yet, this could not be implemented due to the unavailability of phase III and comparative studies. Therefore, conclusions are based on pooled proportion data from available single-arm and retrospective studies.

## 5. Conclusions

This systematic review and meta-analysis of 14 studies and over 200 patients with relapsed/refractory Ewing sarcoma support the clinical activity of TKIs in this setting. The ORR and DCR were 23% and 61%, respectively, in the overall cohort. The overall median PFS was 4.7 months. Among the FDA-approved options, single-agent cabozantinib and regorafenib seem to be the frontrunners. Nonetheless, there are important differences in study design and population that limit our interpretation of efficacy and toxicity findings. On the latter, several studies did not report data exclusively for Ewing sarcoma patients; thus, conclusions about toxicity are mostly based on the general population of studies and may not be fully representative of Ewing sarcoma patients. Moreover, network meta-analysis would be the most appropriate to determine the comparative efficacy and tolerability of TKIs. However, this was unfeasible as most available evidence derives from single-arm and real-world studies. Future advances in biomarker identification tools may help predict those patients who would benefit from TKIs. Liquid biopsies are a promising strategy in assessing tumor heterogeneity and evolution, and complementary targets that could guide TKI-combined therapies in Ewing sarcoma patients. Biomarker testing at time of relapse could reveal important molecular features, such as angiogenesis-related pathways (e.g., VEGF/VEGFR expression), which could inform treatment choices among the TKI agents available. Liquid biopsies are also a promising strategy in Ewing sarcoma. Future studies using novel therapies, such as antibody–drug conjugates and immunotherapy strategies, may also improve the prognosis of refractory/relapsed Ewing sarcoma patients.

## Figures and Tables

**Table 1:** Characteristics of studies included in this meta-analysis.

Study	Design	Location	Treatment Regimens	Primary TKI Targets	N (Ewing)	Female, N (%)	Age, Median (Range)	TKI Starting Dose	Previous Lines of Therapy, Median
Attia 2023 (SARC024) [15]	Phase II CT	USA	Regorafenib	VEGFR1–3; RAF/BRAF; KIT	30	10 (33)	32 (19–65)	160 mg/d PO on days 1–21 of 28	3
Duffaud 2023 (REGOBONE) [27]	Phase II RCT	France	Regorafenib		23	5 (22)	32 (18–59)	160 mg/d PO on days 1–21 of 28	2
Italiano 2020 (CABONE) [16]	Phase II CT	France	Cabozantinib	MET, VEGFR2	45	14 (31)	33 (IQR 24–45)	Adults 60 mg/d, children (<16 years) 40 mg/m^2^/d PO in cycles of 28 days	2
Kokkali 2023 [28]	Retrospective Cohort	Greece	Cabozantinib		5	1 (20)	31 (18–34)	60 mg/d	2
Soroka 2023 (CanSaRCC) ^†^ [31]	Retrospective Cohort	Canada	Cabozantinib		16	9 (31)	25 (12–61)	REGO—120 mg/d PO on days 1–21 of 28 CABO—Median starting dose of 60 mg/d (range 40–60) PO	2
Liu 2021 * [29]	Retrospective Cohort	China	Anlotinib	VEGFR1–3, FGFR1–3	8	NA	24 (16–68)	12 mg/d PO on days 1–14 of 21	1
Xu 2021 [21]	Phase Ib/II CT	China	Anlotinib + Vincristine + Irinotecan		36	12 (33)	mean ± SD: cohort A—28 ± 9 cohort B—11 ± 3	12 mg/d PO on days 1–14 of 21	NA
Xie 2018 * [22]	Retrospective Cohort	China	Apatinib	VEGFR2	10	NA	25 (9–63)	BSA > 1.5 m^2^—750 mg/d PO BSA < 1.5—500 mg/d PO <10 years—250 mg/d PO	1.5
Wang 2018 [20]	Retrospective Cohort	China	Apatinib Apatinib + Chemo Apatinib + Chemo + surgery		11	5 (46)	18 (10–31)	>10 years—500 mg/d <10 years—250 mg/d	1
Bodea 2022 * [24]	Retrospective Cohort	USA	Sorafenib + Bevacizumab + Cyclophosphamide	VEGFR2–3; RAF/BRAF	14	NA	15 (1–22)	180 mg/m^2^/d PO	3
Yang 2024 * [23]	Retrospective Cohort	China	Fruquintinib alone or in combination	VEGFR1–3	28	47 (38)	21 (5–75)	>10 years—7 mg <10 years—3–5 mg (5 days on and 2 days off)	4
Palmerini 2024 * [30]	Phase II CT	Italy and Spain	Sunitinib + Nivolumab	VEGFR1–3, PDGFR	8	13 (32)	47 (21–74)	37.5 mg PO on the first 14 days	2
Chugh 2009 [25]	Phase II CT	USA	Imatinib	BCR-ABL, KIT	13	5 (38)	NA	BSA < 1.0 m^2^—200 mg/d PO BSA 1.0–1.49 m^2^—400 mg/d PO BSA ≥ 1.5 m^2^—600 mg/d PO	NA
Dela Cruz 2024 [26]	Phase I/II CT	USA	Lenvatinib + Everolimus	VEGFR1–3, FGFR1–4	10	3 (30)	16.5 (3–19)	11 mg/m^2^ PO	2

* Data refers to the total population of the study, not only patients with Ewing sarcoma. ^†^ This study included two Ewing sarcoma patients who received regorafenib; however, due to the small sample size (<5 patients), they were not included in our analyses. BCR-ABL: breakpoint cluster region–Abelson fusion tyrosine kinase; BRAF: B-Raf proto-oncogene serine/threonine kinase; BSA: body surface area; Chemo: chemotherapy; CT: clinical trial; d: day; FGFR: fibroblast growth factor receptor; FGFR1: fibroblast growth factor receptor 1; FGFR2: fibroblast growth factor receptor 2; FGFR3: fibroblast growth factor receptor 3; FGFR4: fibroblast growth factor receptor 4; IQR: interquartile range; KIT (c-KIT): stem cell factor receptor (CD117); MET: hepatocyte growth factor receptor; N: number of patients; NA: not available; PDGFR: platelet-derived growth factor receptor; PO: per os; RAF: rapidly accelerated fibrosarcoma kinase; TKI: tyrosine kinase inhibitor; USA: United States of America; VEGFR: vascular endothelial growth factor receptor; VEGFR1: vascular endothelial growth factor receptor 1; VEGFR2: vascular endothelial growth factor receptor 2; VEGFR3: vascular endothelial growth factor receptor 3.

**Table 2:** Pooled efficacy outcomes of this meta-analysis.

(A) Objective response rate (ORR)			
Outcome/Subgroup	N of Pooled Studies	N of Patients	ORR (95% CI)
Overall ORR	14	247	23% (11.2–37.1)
ORR of pooled clinical trials	7	157	19.5% (3.9–41.4)
ORR of pooled real-world studies	7	90	27.4% (13.3–43.8)
ORR by TKI used			
Regorafenib	2	53	11.3% (3.6–21.7)
Cabozantinib	3	59	21.6% (10.8–34.3)
Apatinib	2	20	55.2% (26.1–82.7)
Anlotinib	2	44	63.8% (40.4–84.5)
Sorafenib	1	14	14.3% (1.8–42.8)
Lenvatinib	1	10	0 (0–30.9)
Fruquintinib	1	28	17.9% (6.1–36.9)
Sunitinib	1	6	0 (0–45.9)
Imatinib	1	13	0 (0–24.7)
ORR considering single-agent TKI only			
Regorafenib	2	53	11.3% (3.6–21.7)
Cabozantinib	3	59	21.6% (10.8–34.3)
Anlotinib	1	8	37.5% (34.8–93.3)
Apatinib	1	10	70% (34.8–93.3)
Imatinib	1	13	0 (0–24.7)
ORR in single-agent TKI vs. combined therapy			
TKI single-agent	8	143	18.7% (6.9–33.6)
TKI-combined therapy	6	104	27.7% (6.9–54.3)
(B) Disease control rate (DCR)			
Outcome/Subgroup	N of Pooled Studies	N of Patients	ORR (95% CI)
Overall DCR	14	224	61.1% (47.3–74.2)
ORR of pooled clinical trials	6	134	53.2% (29.7–76.1)
ORR of pooled real-world studies	7	90	66.8% (55.2–77.6)
DCR by TKI used			
Regorafenib	1	30	60% (40.2–85.7)
Cabozantinib	3	59	64.4% (40.2–85.7)
Apatinib	2	20	80% (58.4–95.9)
Anlotinib	2	44	75.5% (60.8–88.0)
Sorafenib	1	14	78.5% (49.2–95.3)
Lenvatinib	1	10	40% (12.2–73.8)
Fruquintinib	1	28	57.1% (37.2–75.5)
Sunitinib	1	6	50% (11.8–88.2)
Imatinib	1	13	0 (0–24.7)
DCR considering single-agent TKI only			
Regorafenib	1	30	60% (40.6–77.3)
Cabozantinib	3	59	64.4% (40.2–85.7)
Anlotinib	1	8	75% (34.9–99.4)
Apatinib	1	10	80% (44.4–97.5)
Imatinib	1	13	0 (0.0–24.7)
DCR in single-agent TKI vs. combined therapy			
TKI single-agent	7	120	55.1% (29.9–79.1)
TKI-combined therapy	6	104	61.7% (47.3–75.3)

DCR: disease control rate; CI: confidence interval; N: number; ORR: objective response rate; TKI: tyrosine kinase inhibitor.

## Data Availability

Data used in this study is available upon reasonable request to the corresponding author.

## Supplementary Materials

The following supporting information can be downloaded at: https://www.mdpi.com/article/10.3390/cancers18030465/s1, Table S1: Preferred Reporting Items for Systematic Reviews and Meta-Analysis (PRISMA) Checklist for the Manuscript (A), and for the Abstract (B); Table S2: Full search used in each database; Table S3: Outcomes definition and assessment criteria used across studies. CR: complete response; DCR: disease control rate; ORR: objective response rate; PFS: progression-free survival; PR: partial response; RECIST: Response Evaluation Criteria in Solid Tumors; SD: stable disease; Table S4: List of excluded studies after full read; Table S5: Adverse events according to each study. * Data available only for the total population of the study and does not refer exclusively to Ewing sarcoma patients. CTCAE: National Cancer Institute Common Terminology Criteria for Adverse Events; TKI: tyrosine kinase inhibitor; Table S6: Adverse events of studies with data available exclusively for patients with Ewing sarcoma. CTCAE: National Cancer Institute Common Terminology Criteria for Adverse Events; TKI: tyrosine kinase inhibitor; Table S7: Tools and criteria used to assess the quality of studies included in this meta-analysis; Table S8: Risk of bias assessment; Table S9: Leave-one-out for overall ORR and DCR. Proportions of the ORR and DCR leaving each study out are described in the “Proportion” column, followed by the respective 95% confidence interval (CI); Figure S1: Response analyses according to the TKI used. Squares represent proportions for each study, and the horizontal line crossing the squares indicates the 95% confidence interval; diamonds represent the estimated overall effect of the meta-analysis based on a random-effects model. CI: confidence intervals; DCR: disease control rate; ORR: objective response rate; TKI: tyrosine kinase inhibitors; Figure S2: Response analyses including only studies on single-agent TKI. Squares represent proportions for each study, and the horizontal line crossing the squares indicates the 95% confidence interval; diamonds represent the estimated overall effect of the meta-analysis based on a random-effects model. CI: confidence intervals; DCR: disease control rate; ORR: objective response rate; TKI: tyrosine kinase inhibitors; Figure S3: Responses stratified per studies on single-agent TKI versus TKI-combined regimens. Squares represent proportions for each study, and the horizontal line crossing the squares indicates the 95% confidence interval; diamonds represent the estimated overall effect of the meta-analysis based on a random-effects model. CI: confidence intervals; DCR: disease control rate; ORR: objective response rate; TKI: tyrosine kinase inhibitors; Figure S4: Median progression-free survival (mPFS) per TKI agent used. Squares represent mPFS for each study, and the horizontal line crossing the squares indicates the 95% confidence interval; diamonds represent the estimated overall effect of the meta-analysis based on a random-effects model. CI: confidence intervals; mPFS: median progression-free survival; TKI: tyrosine kinase inhibitors; Figure S5: Meta-regression on the impact of the median number of prior lines of therapy on the ORR. the dots represent individual studies, the median number of previous therapies is shown on the x-axis, and the response rate on the y-axis; the line indicates the tendency of responses according to the number of previous therapies. ORR: objective response rate.

## Abbreviations

The following abbreviations are used in this manuscript: AEs, adverse events; CI, confidence interval; CT, clinical trial; DCR, disease control rate; DR5, death receptor 5; EFS, event-free survival; Ewing sarcoma, Ewing sarcoma; FDA, Food and Drug Administration; FGFR, fibroblast growth factor receptor; IFOS, ifosfamide; IT, irinotecan–temozolomide; JBI, Joanna Briggs Institute; KIT, stem cell factor receptor; mPFS, median progression-free survival; MINORS, Methodological Index for Non-Randomized Studies; NCI, National Cancer Institute; ORR, objective response rate; OS, overall survival; PDGFR, platelet-derived growth factor receptor; PFS, progression-free survival; PRISMA, Preferred Reporting Items for Systematic Reviews and Meta-Analyses; PROSPERO, International Prospective Register of Systematic Reviews; rEECur, randomized Ewing sarcoma recurrence trial; SE, standard error; TKI, tyrosine kinase inhibitor; VEGF, vascular endothelial growth factor.
