# Supplementary material for: Multi-Targeted TKIs in Patients with Advanced Ewing Sarcoma: A Systematic Review and Single-Arm Meta-Analysis
Source: Cancers (Basel). 2026 Jan 30;18(3):465. doi: 10.3390/cancers18030465 (PMC12896480; doi:10.3390/cancers18030465)
Supplement: Supplementary file 1 [file cancers-18-00465-s001.zip › cancers-4030304-supplementary.pdf]

# Supplementary Material

## Multi-targeted TKIs in Patients with Advanced Ewing Sarcoma: A Systematic Review and Single-Arm Meta-Analysis

Michelon *et al.*

## Table of Contents

**Table S1: Preferred Reporting Items for Systematic Reviews and Meta-Analysis (PRISMA) Checklist for the Manuscript (A), and for the Abstract (B). ..............................................3**

**(A) Manuscript Prisma Checklist ........................................................................ 3**

**(B) Abstract Prisma Checklist ............................................................................. 6**

**Table S2: Full search used in each database. .......................................................................7**

**Table S3: Outcomes definition and assessment criteria used across studies......................8**

**Table S4: List of excluded studies after full read...............................................................10**

**Table S5: Adverse events according to each study. ...........................................................11**

**Table S6: Adverse events of studies with data available exclusively for patients with Ewing sarcoma. ..........................................................................................................................13**

**Table S7: Tools and criteria used to assess the quality of studies included in this meta-analysis. ..........................................................................................................................14**

**Table S8: Risk of bias assessment.........................................................................................16**

**Table S9: Leave-one-out for overall ORR and DCR. .........................................................18**

**Figure S1: Response analyses according to the TKI used. ................................................19**

**Figure S2: Response analyses including only studies on single-agent TKI. .....................20**

**Figure S3: Responses stratified per studies on single-agent TKI versus TKI-combined regimens. ..........................................................................................................................21**

**Figure S4: Median progression-free survival (mPFS) per TKI agent used. ....................22**

**Figure S5: Meta-regression on the impact of the median number of prior lines of therapy on the ORR. ..........................................................................................................................23**

# Tables

## Table S1: Preferred Reporting Items for Systematic Reviews and Meta-Analysis (PRISMA) Checklist for the Manuscript (A), and for the Abstract (B).

### (A) Manuscript Prisma Checklist

| Section and Topic | Item # | Checklist item | Location where item is reported |
|---|---|---|---|
| **TITLE** | | | |
| **Title** | **1** | Identify the report as a systematic review. | **Page 1** |
| **ABSTRACT** | | | |
| **Abstract** | **2** | See the PRISMA 2020 for Abstracts checklist. | **Table S1B** |
| **INTRODUCTION** | | | |
| **Rationale** | **3** | Describe the rationale for the review in the context of existing knowledge. | **Pages 4, 5** |
| **Objectives** | **4** | Provide an explicit statement of the objective(s) or question(s) the review addresses. | **Page 5** |
| **METHODS** | | | |
| **Eligibility criteria** | **5** | Specify the inclusion and exclusion criteria for the review and how studies were grouped for the syntheses. | **Page 5, 6** |
| **Information sources** | **6** | Specify all databases, registers, websites, organizations, reference lists and other sources searched or consulted to identify studies. Specify the date when each source was last searched or consulted. | **Page 6** |
| **Search strategy** | **7** | Present the full search strategies for all databases, registers and websites, including any filters and limits used. | **Table S2** |
| **Selection process** | **8** | Specify the methods used to decide whether a study met the inclusion criteria of the review, including how many reviewers screened each record and each report retrieved, whether they worked independently, and if applicable, details of automation tools used in the process. | **Page 6** |
| **Data collection process** | **9** | Specify the methods used to collect data from reports, including how many reviewers collected data from each report, whether they worked independently, any processes for obtaining or confirming data from study investigators, and if applicable, details of automation tools used in the process. | **Page 6** |
| **Data items** | **10a** | List and define all outcomes for which data were sought. Specify whether all results that were compatible with each outcome domain in each study were sought (e.g. for all measures, time points, analyses), and if not, the methods used to decide which results to collect. | **Page 6** |
| | **10b** | List and define all other variables for which data were sought (e.g. participant and intervention characteristics, funding sources). Describe any assumptions made about any missing or unclear information. | **Page 6** |

| | | | |
|---|---|---|---|
| **Study risk of bias assessment** | **11** | Specify the methods used to assess risk of bias in the included studies, including details of the tool(s) used, how many reviewers assessed each study and whether they worked independently, and if applicable, details of automation tools used in the process. | **Page 4** |
| **Effect measures** | **12** | Specify for each outcome the effect measure(s) (e.g. risk ratio, mean difference) used in the synthesis or presentation of results. | **Page 5** |
| **Synthesis methods** | **13a** | Describe the processes used to decide which studies were eligible for each synthesis (e.g. tabulating the study intervention characteristics and comparing against the planned groups for each synthesis (item #5)). | **Page 6** |
| | **13b** | Describe any methods required to prepare the data for presentation or synthesis, such as handling of missing summary statistics, or data conversions. | **Page 7** |
| | **13c** | Describe any methods used to tabulate or visually display results of individual studies and syntheses. | **Page 7** |
| | **13d** | Describe any methods used to synthesize results and provide a rationale for the choice(s). If meta-analysis was performed, describe the model(s), method(s) to identify the presence and extent of statistical heterogeneity, and software package(s) used. | **Page 7** |
| | **13e** | Describe any methods used to explore possible causes of heterogeneity among study results (e.g. subgroup analysis, meta-regression). | **Pages 6, 7** |
| | **13f** | Describe any sensitivity analyses conducted to assess robustness of the synthesized results. | **Pages 6, 7** |
| **Reporting bias assessment** | **14** | Describe any methods used to assess risk of bias due to missing results in a synthesis (arising from reporting biases). | **NA** |
| **Certainty assessment** | **15** | Describe any methods used to assess certainty (or confidence) in the body of evidence for an outcome. | **NA** |
| RESULTS | | | |
| **Study selection** | **16a** | Describe the results of the search and selection process, from the number of records identified in the search to the number of studies included in the review, ideally using a flow diagram. | **Page 7** |
| | **16b** | Cite studies that might appear to meet the inclusion criteria, but which were excluded, and explain why they were excluded. | **Table S3** |
| **Study characteristics** | **17** | Cite each included study and present its characteristics. | **Table 1** |
| **Risk of bias in studies** | **18** | Present assessments of risk of bias for each included study. | **Table S4** |

| | | | |
|---|---|---|---|
| **Results of individual studies** | **19** | For all outcomes, present, for each study: (a) summary statistics for each group (where appropriate) and (b) an effect estimate and its precision (e.g. confidence/credible interval), ideally using structured tables or plots. | **Figures 2, 3** |
| **Results of syntheses** | **20a** | For each synthesis, briefly summarise the characteristics and risk of bias among contributing studies. | **Table S4** |
| | **20b** | Present results of all statistical syntheses conducted. If meta-analysis was done, present for each the summary estimate and its precision (e.g. confidence/credible interval) and measures of statistical heterogeneity. If comparing groups, describe the direction of the effect. | **Figures 2, 3** |
| | **20c** | Present results of all investigations of possible causes of heterogeneity among study results. | **Page 9** |
| | **20d** | Present results of all sensitivity analyses conducted to assess the robustness of the synthesized results. | **Page 9** |
| **Reporting biases** | **21** | Present assessments of risk of bias due to missing results (arising from reporting biases) for each synthesis assessed. | **NA** |
| **Certainty of evidence** | **22** | Present assessments of certainty (or confidence) in the body of evidence for each outcome assessed. | **NA** |
| DISCUSSION | | | |
| **Discussion** | **23a** | Provide a general interpretation of the results in the context of other evidence. | **Page 9** |
| | **23b** | Discuss any limitations of the evidence included in the review. | **Page 12** |
| | **23c** | Discuss any limitations of the review processes used. | **Page 12** |
| | **23d** | Discuss implications of the results for practice, policy, and future research. | **Page 12** |
| OTHER INFORMATION | | | |
| **Registration and protocol** | **24a** | Provide registration information for the review, including register name and registration number, or state that the review was not registered. | **Page 5** |
| | **24b** | Indicate where the review protocol can be accessed, or state that a protocol was not prepared. | **Page 5** |
| | **24c** | Describe and explain any amendments to information provided at registration or in the protocol. | **NA** |
| **Support** | **25** | Describe sources of financial or non-financial support for the review, and the role of the funders or sponsors in the review. | **Page 14** |
| **Competing interests** | **26** | Declare any competing interests of review authors. | **Page 14** |

| | | | |
|---|---|---|---|
| **Availability of data, code and other materials** | **27** | Report which of the following are publicly available and where they can be found: template data collection forms; data extracted from included studies; data used for all analyses; analytic code; any other materials used in the review. | **Page 14** |

NA: not available

## (B) Abstract Prisma Checklist

| Section and Topic | Item # | Checklist item | Reported (Yes/No) |
|---|---|---|---|
| **TITLE** | | | |
| **Title** | **1** | Identify the report as a systematic review. | **Yes** |
| BACKGROUND | | | |
| **Objectives** | **2** | Provide an explicit statement of the main objective(s) or question(s) the review addresses. | **Yes** |
| METHODS | | | |
| **Eligibility criteria** | **3** | Specify the inclusion and exclusion criteria for the review. | **Yes** |
| **Information sources** | **4** | Specify the information sources (e.g. databases, registers) used to identify studies and the date when each was last searched. | **No** |
| **Risk of bias** | **5** | Specify the methods used to assess risk of bias in the included studies. | **No** |
| **Synthesis of results** | **6** | Specify the methods used to present and synthesise results. | **No** |
| RESULTS | | | |
| **Included studies** | **7** | Give the total number of included studies and participants and summarise relevant characteristics of studies. | **Yes** |
| **Synthesis of results** | **8** | Present results for main outcomes, preferably indicating the number of included studies and participants for each. If meta-analysis was done, report the summary estimate and confidence/credible interval. If comparing groups, indicate the direction of the effect (i.e. which group is favoured). | **Yes** |
| DISCUSSION | | | |
| **Limitations of evidence** | **9** | Provide a brief summary of the limitations of the evidence included in the review (e.g. study risk of bias, inconsistency and imprecision). | **No** |
| **Interpretation** | **10** | Provide a general interpretation of the results and important implications. | **Yes** |
| OTHER | | | |
| **Funding** | **11** | Specify the primary source of funding for the review. | **No** |
| **Registration** | **12** | Provide the register name and registration number. | **No** |

**Table S2: Full search used in each database.**

| | |
|---|---|
| **PubMed** | Ewing AND ("tyrosine kinase inhibitor" OR TKI OR regorafenib OR cabozantinib OR anlotinib OR sorafenib OR lenvatinib OR fruquintinib OR sunitinib OR apatinib OR imatinib OR pazopanib) |
| **Cochrane** | Ewing AND ("tyrosine kinase inhibitor" OR TKI OR regorafenib OR cabozantinib OR anlotinib OR sorafenib OR lenvatinib OR fruquintinib OR sunitinib OR apatinib OR imatinib OR pazopanib) |
| **Embase** | ewing:ab,ti AND ('tyrosine kinase inhibitor':ab,ti OR tki:ab,ti OR regorafenib:ab,ti OR cabozantinib:ab,ti OR anlotinib:ab,ti OR sorafenib:ab,ti OR lenvatinib:ab,ti OR fruquintinib:ab,ti OR sunitinib:ab,ti OR apatinib:ab,ti OR imatinib:ab,ti OR pazopanib:ab,ti) |

**Table S3: Outcomes definition and assessment criteria used across studies.**

| Study | ORR definition | DCR definition | PFS definition | Scale |
|---|---|---|---|---|
| **Attita 2023 (SARC024)** | percentage of patients whose cancer shrinks or disappears after treatment | percentage of patients who achieved ORR or stable disease on treatment | time to treatment start to progression or death | RECIST version 1.1 |
| **Duffaud 2023 (REGOBONE)** | CR or PR as best response from randomisation | NA | date of randomisation to confirmed radiological progression or death | RECIST version 1.1 |
| **Italiano 2020 (CABONE)** | PR as best response within 6 months, assessed by RECIST v1.1, based on blinded central radiological review | best overall response defined as CR, PR, or SD, assessed from treatment start until progression, per RECIST v1.1, central review | time from treatment onset to progression or death from any cause, whichever occurred first; censored at last follow-up | RECIST version 1.1 |
| **Kokkali 2023** | CR or PR, assessed according to RECIST v1.1 | SD as best response; among evaluable ES patients | time from cabozantinib initiation to disease progression or death from any cause, assessed per RECIST v1.1. | RECIST v1.1 |
| **Soroka 2023** | CR or PR, assessed by local investigators | CR + PR + SD lasting > 3 months | time from TKI initiation to disease progression or death, assessed by local investigators | NA |
| **Liu 2021** | proportion of patients achieving CR or PR, assessed using RECIST v1.1 | proportion of patients achieving CR, PR or SD, assessed using RECIST v1.1. | time from treatment initiation to disease progression or death, assessed using RECIST v1.1 | RECIST v1.1 |
| **Xie 2018** | CR + PR, assessed according to RECIST v1.1. | proportion of patients achieving CR or PR or SD, assessed using RECIST v1.1. | time from start of apatinib to disease progression or death, whichever occurred first | RECIST v1.1 |
| **Xu 2021** | proportion of patients with CR or PR at 12 weeks, assessed by RECIST v1.1. | proportion of patients with CR or PR or SD at 12 weeks, assessed by RECIST v1.1. | time from study entry until documented progression, protocol violation at investigator discretion, serious | RECIST v1.1 |

| | | | | |
|---|---|---|---|---|
| | | | adverse effect, or death from any cause. | |
| **Wang 2018** | CR or PR, assessed according to RECIST v1.1 | CR + PR + SD, assessed according to RECIST v1.1. | time from apatinib initiation to disease progression or death | RECIST v1.1 |
| **Bodea 2022** | Objective response assessed using RECIST v1.1 (CR or PR as best response) | DCR corresponds to CR + PR + SD | NA | RECIST v1.1 |
| **Yang 2024** | proportion of patients with best overall response of CR or PR, assessed by RECIST v1.1 | proportion of patients with best overall response of CR, PR, or SD, assessed by RECIST v1.1 | time from first fruquintinib administration to documented progression or death, whichever occurred first | RECIST v1.1 |
| **Palmerini 2024** | CR + PR per RECIST v1.1 (central radiology review) | CR + PR + SD per RECIST v1.1 | time from enrollment to progression or death, assessed by RECIST v1.1 with central review | RECIST v1.1 |
| **Chugh 2009** | CR or PR by radiologic assessment | NA | time from treatment initiation to disease progression or death | NA |
| **Dela Cruz 2024** | CR + PR per RECIST v1.1, assessed at Week 16 | CR + PR + stable disease lasting ≥ 7 weeks from first dose. | NA | RECIST v1.1 |

CR: complete response, DCR: disease control rate; ORR: objective response rate; PFS: progression-free survival; PR: partial response; RECIST: Response Evaluation Criteria in Solid Tumors; SD: stable disease.

**Table S4: List of excluded studies after full read.**

| Author | TKI | Exclusion reason |
|---|---|---|
| Akshintala 2021 | Cabozantinib | Unpublished (abstract only available). No Ewing subgroup analysis. |
| Campbell 2023 | Cabozantinib | Phase I trial. |
| Geoerger 2021 | Regorafenib | Phase I trial. No Ewing subgroup analysis. |
| Bond 2008 | Imatinib | No outcomes of interest. No Ewing sarcoma subgroup analysis. |
| Tang 2022 | Anlotinib | <5 Ewing sarcoma patients. |
| Chuk 2018 | Cabozantinib | Phase I trial. <5 Ewing sarcoma patients. |
| Garnier 2016 | Targeted therapies | No targeted therapies subgroup analysis. No Ewing subgroup analyis. |
| Geller 2018 | Axitinib | Phase I trial. <5 Ewing sarcoma patients. |
| Chen 2018 | Apatinib | Unpublished (abstract only available). <5 Ewing sarcoma patients. |
| Raciborska 2018 | Sorafenib | <5 Ewing sarcoma patients. |
| Gaspar 2017 | Lenvatinib | No Ewing subgroup analysis. |
| Aggerholm 2020 | Pazopanib | <5 Ewing sarcoma patients. |
| Seto 2019 | Pazopanib | <5 Ewing sarcoma patients. |
| Subbiah 2017 | Pazopanib | <5 Ewing sarcoma patients. |
| Wang 2020 | Anlotinib | No Ewing subgroup analysis. |

**Table S5: Adverse events according to each study.**

| Study | TKI | Toxicity assessment criteria | N | Most common AEs | Grade 1-2<br>N (%) | Grade 3-4<br>N (%) | Dose reductions<br>N (%) | Treatment discontinuation<br>N (%) |
|---|---|---|---|---|---|---|---|---|
| **Attia 2023/SARC024** | Regorafenib | CTCAE v4.03 | 30 | Hypophosphatemia<br>Hypertension<br>Transaminasemia | 0<br>3 (10%)<br>0 | 6 (20%)<br>2 (6.7%)<br>2 (6.7%) | 16 (53.3%) | 2 (6.6%) |
| **Bodea 2022*** | Sorafenib | CTCAE | 37 | Lymphopenia<br>Leucopenia<br>Palmar-plantar erythrodysesthesia | NA | NA | 33 (63.9%) | 2 (5.1%) |
| **Chugh 2009** | Imatinib | CTCAE v2.0 | 13 | NA | NA | NA | NA | NA |
| **Dela Cruz 2024** | Fruquitinib | CTCAE v4.03 | 10 | Leucopenia<br>Proteinuria<br>Lymphopenia | 5 (50%)<br>5 (50%)<br>2 (20%) | 1 (10%)<br>1 (10%)<br>3 (30%) | NA | 3 (7.3%) |
| **Duffaud 2023 (REGOBONE)** | Regorafenib | CTCAE v4.0 | 23 | Pain<br>Asthenia<br>Diarrhea | 12 (52.2%)<br>12 (52.2%)<br>11 (47.8%) | 5 (27.1%)<br>4 (17.4%)<br>3 (13%) | 9 (39.1%) | NA |
| **Italiano 2020 (CABONE)** | Cabozantinib | CTCAE v4.0 | 45 | Fatigue<br>Diarrhea<br>Mucositis oral | 26 (57.8%)<br>23 (51.1%)<br>24 (53.3%) | 3 (6.7%)<br>2 (4.4%)<br>1 (2.2%) | 19 (21%) | 12 (14.0%) |
| **Kokkali 2023*** | Cabozantinib | NA | 16 | Fatigue<br>Anorexia<br>Transaminasemia | 5 (31.3%)<br>3 (18.8%)<br>3 (18.8%) | NA | 5 (31.3%) | 2 (12.5%) |
| **Liu 2021*** | Anlotinib | CTCAE v4.0 | 48 | Hand-foot syndrome<br>Cholesterol elevation<br>Hypertrigliceridemia | 28 (58.3%)<br>20 (41.6%)<br>18 (37.5%) | 2 (4.2%)<br>1 (2.1%)<br>1 (2.1%) | 8 (16.7%) | 2 (4.2%) |
| **Palmerini 2024*** | Sunitinib | CTCAE v4.0 | 40 | Hypertension<br>Fatigue<br>Diarrhea<br>Mucositis | 19 (47.5%)<br>22 (55.0%)<br>28 (45.0%)<br>16 (40.0%) | 6 (15.0%)<br>3 (7.5%)<br>0<br>2 (5.0%) | 1 (2.5%) | 7 (17.0%) |
| **Soroka 2023 (CanSaRCC)** | Regorafenib | CTCAE v5.0 | 29 | NA | NA | NA | 13 (44.8%) | NA |
| **Soroka 2023 (CanSaRCC)** | Cabozantinib | CTCAE v5.0 | 37 | NA | NA | NA | 15 (40.5%) | NA |
| **Wang 2018** | Apatinib | CTCAE | 10 | Hand-foot syndrome<br>Oral ulcers<br>Gastrointestinal disconfort | 5 (50%)<br>4 (40%)<br>4 (40%) | 1 (10%)<br>0<br>0 | NA | 1 (10.0%) |

| | | | | | | | | |
|---|---|---|---|---|---|---|---|---|
| **Xie 2018*** | Apatinib | CTCAE | 45 | Hypertension<br>Rash<br>Hair hypopigmentation | 30 (67.7%)<br>24 (53.3%)<br>25 (55.6%) | 5 (11.1%)<br>2 (0.4%)<br>0 | NA | 10 (18.0%) |
| **Xu 2021** | Anlotinib | CTCAE v4.0 | 60 | NA | NA | NA | NA | NA |
| **Yang 2024*** | Fruquitinib | CTCAE v5.0 | 124 | Pneumothorax<br>Diarrhea<br>Thrombocytopenia | 0<br>1 (0.8%)<br>1 (0.8%) | 18 (14.5%)<br>7 (5.6%)<br>6 (4.8%) | NA | NA |

*Data available only for the total population of the study and does not refer exclusively to Ewing sarcoma patients. CTCAE: National Cancer Institute Common Terminology Criteria for Adverse Events; TKI: tyrosine kinase inhibitor.

**Table S6: Adverse events of studies with data available exclusively for patients with Ewing sarcoma.**

| Study | TKI | N | Most common AEs | Grade 1-2 N (%) | Grade 3-4 N (%) | Dose reductions N (%) | Treatment discontinuation N (%) |
|---|---|---|---|---|---|---|---|
| **Attia 2023/SARC024** | Regorafenib | 30 | Hypophosphatemia (n=6/30; 20%) | 0 | 6 (20%) | 16 (53.3%) | 2 (6.6%) |
| | | | Hypertension (n=5/30) | 3 (10%) | 2 (6.7%) | | |
| | | | Transaminasemia (n=2/30) | 0 | 2 (6.7%) | | |
| **Dela Cruz 2024** | Fruquitinib | 10 | Leucopenia (n=6/10; 60%) | 5 (50%) | 1 (10%) | NA | 3 (7.3%) |
| | | | Proteinuria (n=6/10; 60%) | 5 (50%) | 1 (10%) | | |
| | | | Lymphopenia (n=5/10; 50%) | 2 (20%) | 3 (30%) | | |
| **Duffaud 2023 (REGOBONE)** | Regorafenib | 23 | Pain (n=17/23; 73.9%) | 12 (52.2%) | 5 (27.1%) | 9 (39.1%) | NA |
| | | | Asthenia (n=16/23; 69.9%) | 12 (52.2%) | 4 (17.4%) | | |
| | | | Diarrhea (n=14/23; 60.9%) | 11 (47.8%) | 3 (13%) | | |
| **Italiano 2020 (CABONE)** | Cabozantinib | 45 | Fatigue (n=29/45; 64.4%) | 26 (57.8%) | 3 (6.7%) | 19 (21%) | 12 (14.0%) |
| | | | Diarrhea (n=25/45; 55.5%) | 23 (51.1%) | 2 (4.4%) | | |
| | | | Mucositis oral (n=25/45; 55.5%) | 24 (53.3%) | 1 (2.2%) | | |
| **Wang 2018** | Apatinib | 10 | Hand-foot syndrome | 5 (50%) | 1 (10%) | NA | 1 (10.0%) |
| | | | Oral ulcers | 4 (40%) | 0 | | |
| | | | Gastrointestinal disconfort | 4 (40%) | 0 | | |

CTCAE: National Cancer Institute Common Terminology Criteria for Adverse Events; TKI: tyrosine kinase inhibitor.

**Table S7: Tools and criteria used to assess the quality of studies included in this meta-analysis.**

**(A) Methodological items for non-randomized studies (MINORS) tool:**

| |
|---|
| I. A clearly stated aim: Is the research question clear, precise, and relevant? |
| II. Inclusion of consecutive patients: Have all eligible patients been included during the study period? |
| III. Prospective collection of data: Was data collected according to prespecified criteria described in the protocol? |
| IV. Endpoints appropriate to the aim of the study: Were endpoints and definitions clearly defined and presented per protocol? |
| V. Unbiased assessment of the study endpoint: Were outcomes evaluated through blind assessment of objective endpoints? If not, were reseasons provided? |
| VI. Follow-up appropriate to the aim of the study: Was the follow-up time adequate? |
| VII. Loss to follow-up less than 5%: Were all patients included in the follow-up? Otherwise, the proportion lost to follow-up should not exceed the proportion experiencing the major endpoint. |
| VIII. Prospective calculation of study size: Has the calculation of sample size been adequately explained and conducted using adequate statistical methods? |

Items are scored 0 (not reported), 1 (reported but inadequate), or 2 (reported and adequate).

- Good quality/low risk of bias – 15-16 points
- Moderate quality and risk of bias – 9-14 points
- Poor quality and high risk of bias – $\leq 8$

**(B) Joanna Briggs Institute Critical Appraisal Tool for Cohort Studies adapted for non-comparative cohort studies**

| |
|---|
| 1. Were all participants recruited according to clear, pre-specified eligibility criteria from a well-defined (and representative) source population? |
| 2. Was the intervention/exposure (e.g. TKI treatment) defined and measured in a consistent, reliable way for every participant? |
| 3. Was the exposure measured in a valid and reliable way? |
| 4. Were confounding factors identified? |
| 5. Were strategies to deal with confounding factors stated? |
| 6. Were the groups/participants free of the outcome at the start of the study? |
| 7. Were the outcomes measured in a valid and reliable way? |
| 8. Was the follow-up time reported and sufficient? |
| 9. Was follow-up complete, and if not, were reasons for loss described? |
| 10. Were strategies to address incomplete follow-up utilized? |
| 11. Was appropriate statistical analysis used? |

Questions are answered as "yes", "no" or "unclear"

- Low risk – If 'yes' to all questions
- Moderate risk – If 'no' for one of the questions 4, 5, and/or 6
- High risk – If 'no' for more than one of the questions 4, 5, and/or 6, or if 'no' for one of these questions and any other

**Table S8: Risk of bias assessment**

(A) MINORS

| MINORS Index for included prospective non-randomized studies | | | | | | | | | |
|---|---|---|---|---|---|---|---|---|---|
| **Study** | **I** | **II** | **III** | **IV** | **V** | **VI** | **VII** | **VIII** | **Total** |
| Attia 2023 (SARC024) | 2 | 2 | 2 | 2 | 1 | 2 | 2 | 2 | 15 - low risk |
| Chugh 2009 | 2 | 2 | 2 | 2 | 0 | 2 | 2 | 2 | 14 - moderate risk |
| Dela Cruz 2025 | 2 | 2 | 2 | 2 | 0 | 2 | 2 | 2 | 14 - moderate risk |
| Duffaud 2023 (REGOBONE) | 2 | 2 | 2 | 2 | 2 | 2 | 2 | 2 | 16 - low risk |
| Italiano 2020 (CABONE) | 2 | 2 | 2 | 2 | 2 | 2 | 2 | 2 | 16 - low risk |
| Palmerini 2024 | 2 | 2 | 2 | 2 | 0 | 2 | 2 | 2 | 14 - moderate risk |
| Xu 2021 | 2 | 2 | 2 | 2 | 0 | 2 | 2 | 2 | 14 - moderate risk |

(B) JBI

| JBI Critical Appraisal Checklist for Cohort Studies | | | | | | | | | | | | |
|---|---|---|---|---|---|---|---|---|---|---|---|---|
| **Study** | **Q1** | **Q2** | **Q3** | **Q4** | **Q5** | **Q6** | **Q7** | **Q8** | **Q9** | **Q10** | **Q11** | **Overall Appraisal** |
| Bodea 2022 | NA | NA | Yes | Yes | No | No | Yes | Yes | Yes | Yes | Yes | Moderate risk |
| Kokkali 2023 | NA | NA | Yes | Yes | No | No | Yes | Yes | Yes | Yes | Yes | Moderate risk |
| Liu 2021 | NA | NA | Yes | Yes | No | No | Yes | Yes | Yes | Yes | Yes | Moderate risk |
| Soroka 2023 | NA | NA | Yes | Yes | Yes | No | Yes | Yes | Yes | Yes | Yes | Moderate risk |
| Wang 2018 | NA | NA | Yes | Yes | No | No | Yes | Yes | Yes | Yes | Yes | Moderate risk |
| Xie 2018 | NA | NA | Yes | Yes | Yes | No | Yes | No | No | Yes | Yes | Moderate risk |
| Yang 2024 | NA | NA | Yes | Yes | Yes | No | Yes | Yes | Yes | Yes | Yes | Moderate risk |

**Table S9: Leave-one-out for overall ORR and DCR.**

**(A) Objective response rate (ORR)**

| Omitted study | Proportion (%) | 95% CI (%) | $\tau^2$ | $\tau$ | $I^2$ |
|---|---|---|---|---|---|
| Attia 2023 (SARC024) | 24.35 | 11.46–39.62 | 0.0618 | 0.2486 | 79.4% |
| Duffaud 2023 (REGOBONE) | 23.93 | 11.05–39.26 | 0.0634 | 0.2518 | 80.3% |
| Kokkali 2023 | 24.58 | 12.26–39.05 | 0.0585 | 0.2420 | 80.4% |
| Italiano 2020 (CABONE) | 22.75 | 9.67–38.63 | 0.0700 | 0.2645 | 80.8% |
| Soroka 2023 | 22.84 | 10.23–38.01 | 0.0637 | 0.2523 | 80.8% |
| Xie 2018 | 20.34 | 9.18–33.89 | 0.0521 | 0.2282 | 78.0% |
| Wang 2018 | 21.97 | 9.84–36.62 | 0.0606 | 0.2461 | 80.5% |
| Liu 2021 | 22.20 | 10.07–36.82 | 0.0606 | 0.2461 | 80.6% |
| Xu 2021 (Cohort A) | 20.10 | 9.23–33.24 | 0.0470 | 0.2168 | 75.0% |
| Xu 2021 (Cohort B) | 19.35 | 9.15–31.64 | 0.0414 | 0.2035 | 73.6% |
| Bodea 2022 | 23.70 | 11.01–38.81 | 0.0623 | 0.2496 | 80.6% |
| Dela Cruz 2025 | 25.41 | 12.95–39.91 | 0.0551 | 0.2347 | 78.9% |
| Yang 2024 | 23.46 | 10.48–39.04 | 0.0662 | 0.2572 | 80.6% |
| Palmerini 2024 | 24.90 | 12.47–39.45 | 0.0576 | 0.2400 | 79.9% |
| Chugh 2021 | 25.69 | 13.31–40.05 | 0.0528 | 0.2299 | 78.0% |
| **Random-effects model (all studies)** | **23.02** | **11.17–37.07** | **0.0580** | **0.2407** | **79.3%** |

**(B) Disease control rate (DCR)**

| Omitted study | Proportion (%) | 95% CI (%) | $\tau^2$ | $\tau$ | $I^2$ |
|---|---|---|---|---|---|
| Attia 2023 (SARC024) | 61.22 | 45.73–75.78 | 0.0485 | 0.2203 | 74.4% |
| Soroka 2023 | 62.57 | 47.92–76.28 | 0.0427 | 0.2066 | 73.2% |
| Kokkali 2023 | 60.47 | 46.22–73.98 | 0.0426 | 0.2063 | 74.3% |
| Italiano 2020 (CABONE) | 59.65 | 44.46–74.08 | 0.0456 | 0.2134 | 72.4% |
| Xie 2018 | 59.70 | 45.14–73.54 | 0.0424 | 0.2059 | 73.6% |
| Wang 2018 | 59.70 | 45.14–73.54 | 0.0424 | 0.2059 | 73.6% |
| Xu 2021 (Cohort A) | 60.16 | 44.98–74.53 | 0.0460 | 0.2145 | 73.9% |
| Liu 2021 | 60.20 | 45.64–74.02 | 0.0430 | 0.2075 | 74.1% |
| Xu 2021 (Cohort B) | 59.27 | 44.79–73.07 | 0.0413 | 0.2032 | 72.9% |
| Bodea 2022 | 59.62 | 44.93–73.59 | 0.0428 | 0.2068 | 73.4% |
| Dela Cruz 2025 | 62.59 | 48.17–76.09 | 0.0417 | 0.2043 | 73.3% |
| Yang 2024 | 61.50 | 46.11–75.94 | 0.0478 | 0.2186 | 74.2% |
| Palmerini 2024 | 61.70 | 47.29–75.26 | 0.0428 | 0.2068 | 74.2% |
| Chugh 2021 | 67.22 | 59.70–74.37 | 0.0017 | 0.0417 | 10.1% |
| **Random-effects model (all studies)** | **61.13** | **47.29–74.22** | **0.0406** | **0.2014** | **72.2%** |

Proportions of the ORR and DCR leaving each study out are described in the "Proportion" column followed by the respective 95% confidence interval (CI).

# Figures

## Figure S1: Response analyses according to the TKI used.

### (A) Objective response rate (ORR)

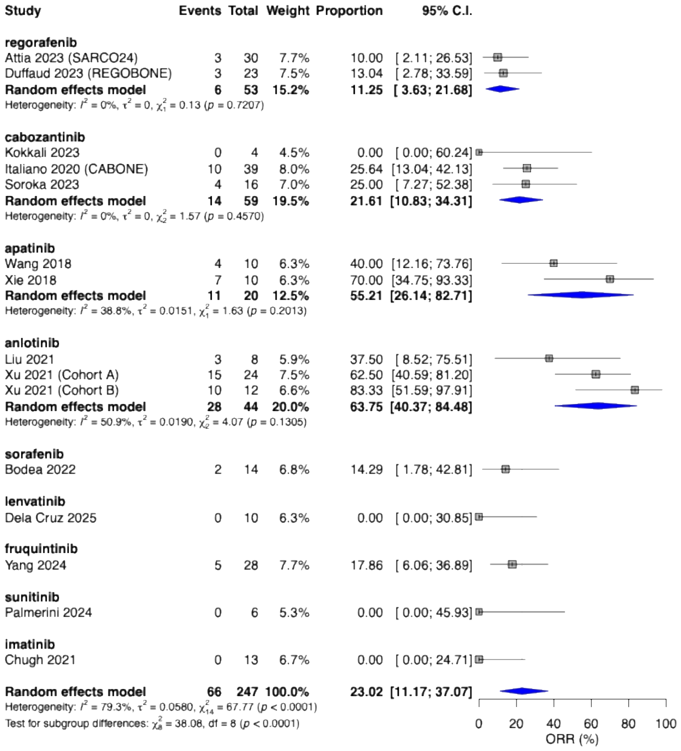


### (B) Disease control rate (DCR)

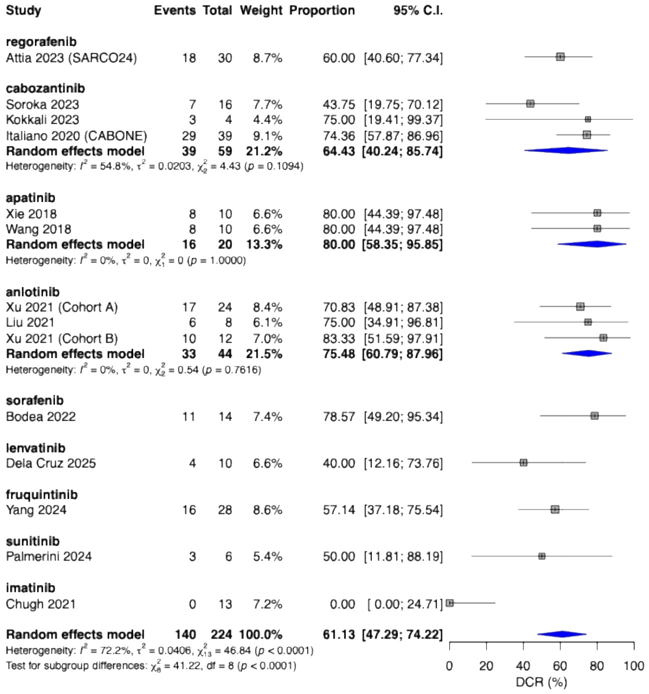


Squares represent proportions for each study, and the horizontal line crossing the squares indicates the 95% confidence interval. Diamonds represent the estimated overall effect of the meta-analysis based on a random-effects model. CI: confidence intervals; DCR: disease control rate; ORR: objective response rate; TKI: tyrosine kinase inhibitors.

**Figure S2: Response analyses including only studies on single-agent TKI.**

**(A) Objective response rate (ORR)**

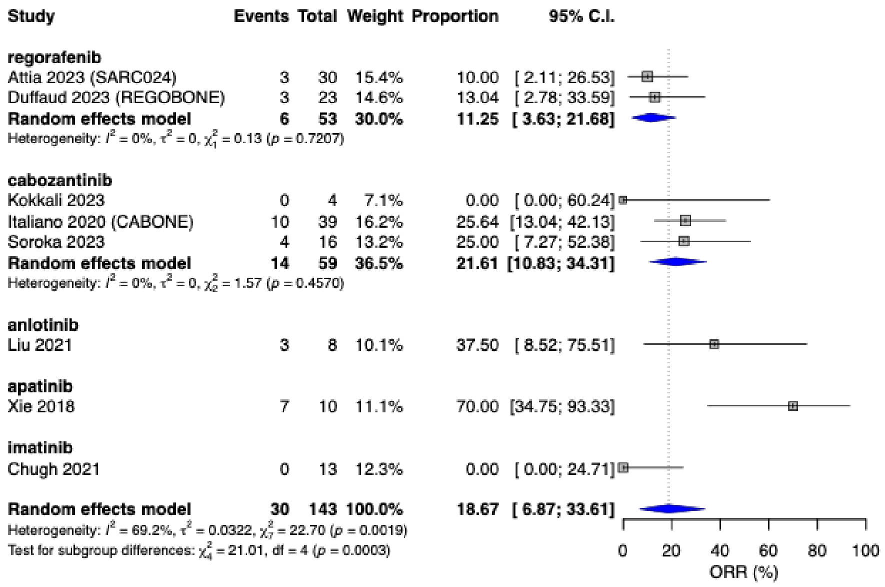


**(B) Disease control rate (DCR)**

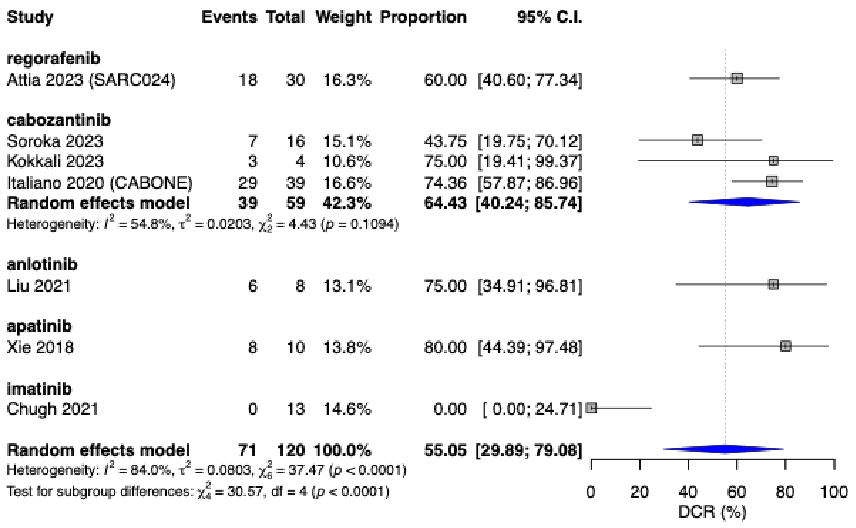


Squares represent proportions for each study, and the horizontal line crossing the squares indicates the 95% confidence interval. Diamonds represent the estimated overall effect of the meta-analysis based on a random-effects model. CI: confidence intervals; DCR: disease control rate; ORR: objective response rate; TKI: tyrosine kinase inhibitors.

## Figure S3: Responses stratified per studies on single-agent TKI *versus* TKI-combined regimens.

### (A) Objective response rate (ORR)

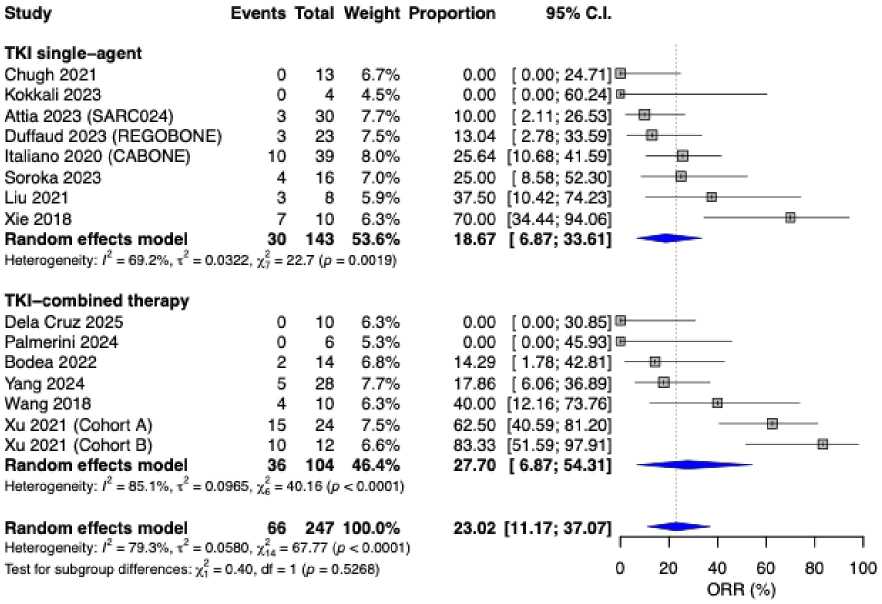


### (B) Disease control rate (DCR)

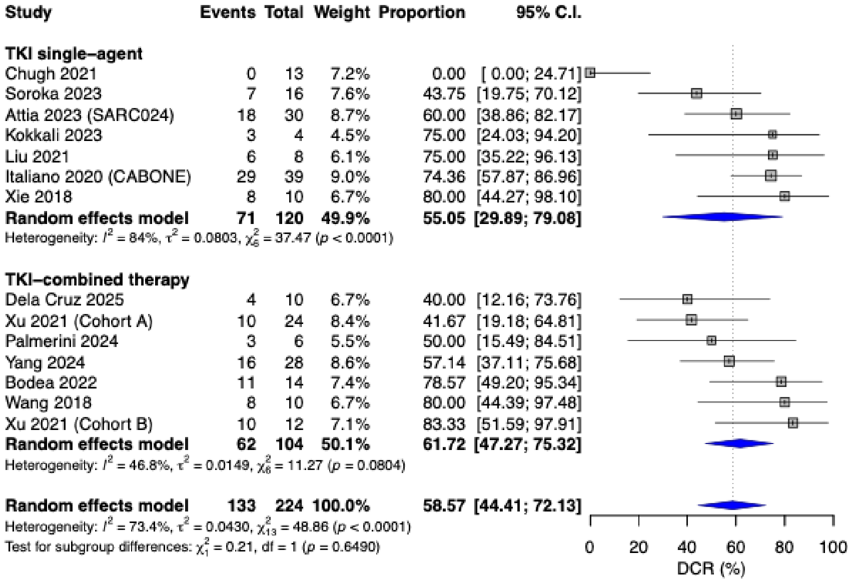


Squares represent proportions for each study, and the horizontal line crossing the squares indicates the 95% confidence interval. Diamonds represent the estimated overall effect of the meta-analysis based on a random-effects model. CI: confidence intervals; DCR: disease control rate; ORR: objective response rate; TKI: tyrosine kinase inhibitors.

## Figure S4: Median progression-free survival (mPFS) per TKI agent used.

| Study | log[mPFS] | SE | mPFS | 95% C.I. | Weight |
|---|---|---|---|---|---|
| **fruquintinib** | | | | | |
| Yang 2024 | 1.0296 | 0.2183 | 2.80 | [1.83; 4.30] | 12.3% |
| **sunitinib** | | | | | |
| Palmerini 2024 | 1.6864 | 0.8336 | 5.40 | [1.05; 27.67] | 2.6% |
| **regorafenib** | | | | | |
| Attia 2023 (SARC024) | 1.2528 | 0.1984 | 3.50 | [2.37; 5.16] | 12.9% |
| Duffaud 2023 (REGOBONE) | 0.9555 | 0.4011 | 2.60 | [1.18; 5.71] | 7.4% |
| **Random effects model** | | | **3.30** | **[2.33; 4.68]** | **20.3%** |
| Heterogeneity: $I^2 = 0\%$, $\tau^2 = 0$, $\chi^2_1 = 0.44$ ($p = 0.5064$) | | | | | |
| **cabozantinib** | | | | | |
| Soroka 2023 | 1.2238 | 0.2337 | 3.40 | [2.15; 5.38] | 11.8% |
| Italiano 2020 (CABONE) | 1.4816 | 0.1057 | 4.40 | [3.58; 5.41] | 15.6% |
| Kokkali 2023 | 1.7405 | 0.2863 | 5.70 | [3.25; 9.99] | 10.2% |
| **Random effects model** | | | **4.34** | **[3.62; 5.20]** | **37.7%** |
| Heterogeneity: $I^2 = 0.7\%$, $\tau^2 = 0.0003$, $\chi^2_2 = 2.01$ ($p = 0.3653$) | | | | | |
| **anlotinib** | | | | | |
| Liu 2021 | 1.9021 | 0.6580 | 6.70 | [1.84; 24.33] | 3.8% |
| Xu 2021 (Cohort A) | 2.3026 | 0.2161 | 10.00 | [6.55; 15.27] | 12.4% |
| Xu 2021 (Cohort B) | 2.0794 | 0.2627 | 8.00 | [4.78; 13.39] | 10.9% |
| **Random effects model** | | | **8.97** | **[6.53; 12.31]** | **27.1%** |
| Heterogeneity: $I^2 = 0\%$, $\tau^2 = 0$, $\chi^2_2 = 0.64$ ($p = 0.7263$) | | | | | |
| **Random effects model** | | | **4.67** | **[3.52; 6.21]** | **100.0%** |
| Heterogeneity: $I^2 = 69.2\%$, $\tau^2 = 0.1233$, $\chi^2_9 = 29.20$ ($p = 0.0006$) | | | | | |
| Test for subgroup differences: $\chi^2_4 = 26.10$, df = 4 ($p < 0.0001$) | | | | | |

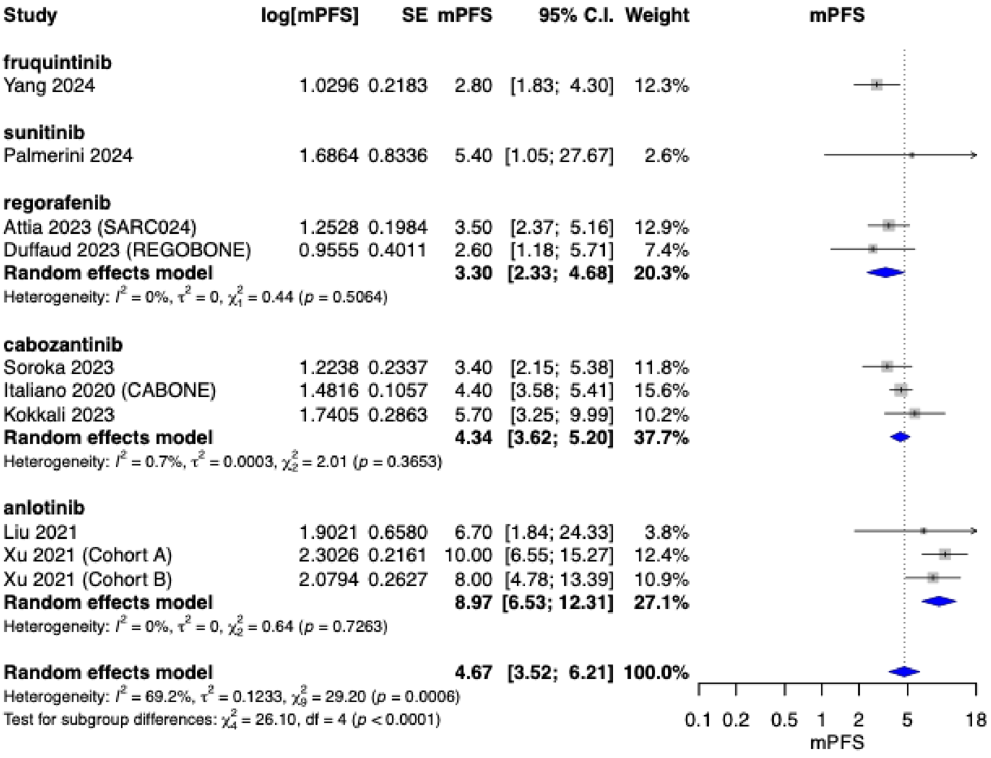


Squares represent mPFS for each study, and the horizontal line crossing the squares indicates the 95% confidence interval. Diamonds represent the estimated overall effect of the meta-analysis based on a random-effects model. CI: confidence intervals; mPFS: median progression-free survival; TKI: tyrosine kinase inhibitors.

**Figure S5: Meta-regression on the impact of the median number of prior lines of therapy on the ORR.**

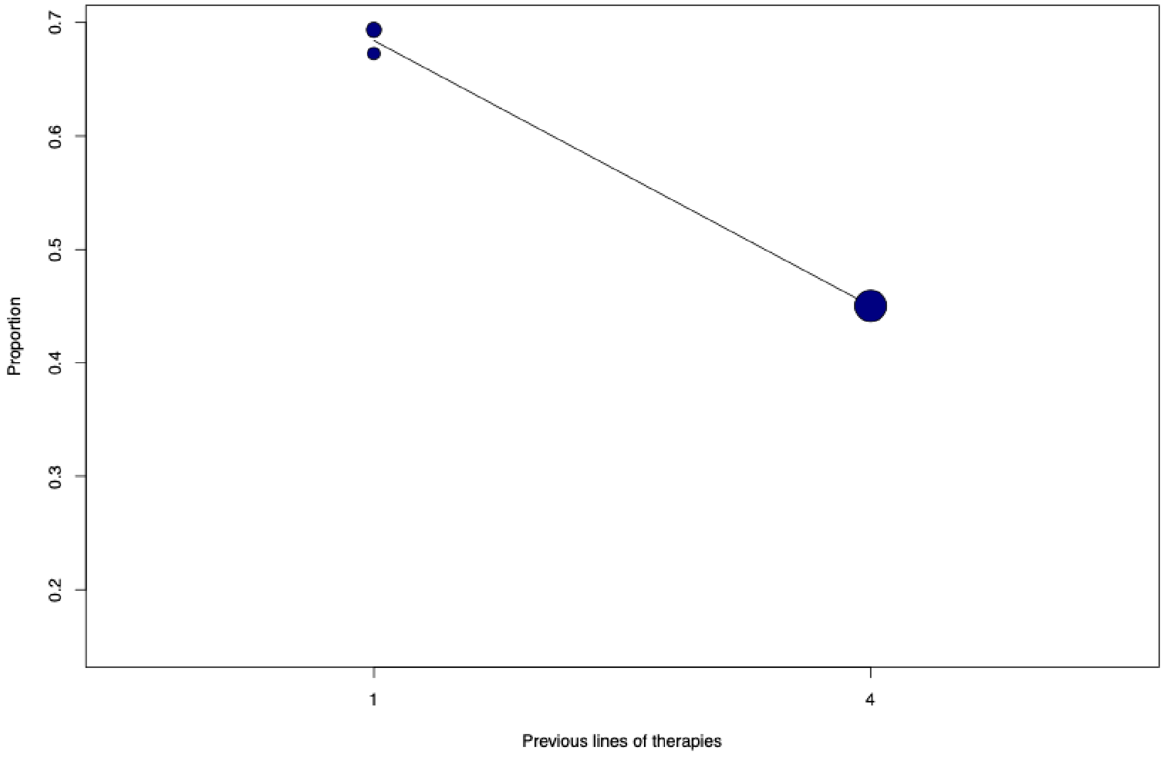


The dots represent individual studies, the median number of previous therapies is shown on the x-axis, and the response rate on the y-axis. The line indicates the tendency of responses according to the number of previous therapies. ORR: objective response rate

| | **Effect estimate** | **p value** | **$I^2$** | **$R^2$** | **p value for residual heterogeneity** |
|---|---|---|---|---|---|
| Intercept | 0.68 | <0.0001 | 19.9% | 13.8% | 0.27 |
| 1.5 vs 1 line | –0.19 | 0.36 | | | |
| 2 lines vs 1 line | –0.28 | **0.04** | | | |
| 3 lines vs 1 line | –0.31 | **0.03** | | | |
| 4 lines vs 1 line | –0.23 | 0.16 | | | |
